# CD44 exerts a functional role during EMT induction in cisplatin-resistant head and neck cancer cells

**DOI:** 10.18632/oncotarget.24252

**Published:** 2018-01-13

**Authors:** Hiroaki Miyazaki, Ryou-u Takahashi, Marta Prieto-Vila, Yumi Kawamura, Seiji Kondo, Tatsuo Shirota, Takahiro Ochiya

**Affiliations:** ^1^ Division of Molecular and Cellular Medicine, National Cancer Center Research Institute, Tokyo 104-0045, Japan; ^2^ Department of Oral and Maxillofacial Surgery, Showa University School of Dentistry, Tokyo 145-8515, Japan; ^3^ Ph.D. Program in Human Biology, School of Integrative and Global Majors, University of Tsukuba 1-1-1 Tennodai, Ibaraki 305-8577, Japan

**Keywords:** oral cancer, CD44, EMT, cancer stem cell niche

## Abstract

A number of studies report that epithelial to mesenchymal transition (EMT) supports the generation and maintenance of cancer stem cells (CSCs), which show tumor seeding ability and drug resistance; however, the molecular mechanisms underlying induction of EMT-associated tumor malignancy remain unclear. The present study reports that oral cancer cells switch from expressing the CD44 variant form (CD44v) to expressing the standard form (CD44s) during acquisition of cisplatin-resistance, which resulted in EMT induction. CD44s induced an EMT phenotype in cisplatin resistant cells by up-regulating ZEB1, a transcriptional repressor of E-cadherin. More importantly, CD44s up-regulated ZEB1 by suppressing microRNA-200c, which is a non-coding RNA that directly represses the ZEB1 gene. These results demonstrate the importance of the association between platinum resistance and CD44s during EMT induction in oral cancer cells.

## INTRODUCTION

Head and neck cancer is one of the most common malignancies, with more than 550,000 cases annually worldwide [1]. In Japan, approximately 8000 people died from head and neck cancer in 2012, accounting for 2.2% of cancer-related deaths [2]. While research has succeeded in improving the quality of life of those with advanced head and neck squamous cell carcinoma (HNSCC), the recurrence and mortality rates remain high [2]. To improve the prognosis of post-operative HNSCC with high-risk features, patients often receive a combination of cisplatin [cis-diamminedichloroplatinum II (CDDP)] and radiotherapy (RT), which provides a survival benefit over RT alone [2]. Therefore, chemo-radiotherapy (CRT) with CDDP is the standard treatment for post-operative HNSCC with a high risk of recurrence.

One of the major obstacles to treatment of cancer patients is acquired resistance to anti-cancer agents [3]. Therefore, the mechanism underlying acquired chemo-resistance is a major topic in current cancer research not only in academic circles but also in clinical practice, particularly with regard to improved therapeutic effects. In HNSCC, as for other types of cancer, intrinsic/acquired resistance to CDDP is a major obstacle, resulting in disease recurrence and a poor prognosis [4].

A number of studies report that HNSCC comprises a heterogeneous cell population; it also shows CSC-like properties such as high tumor seeding ability and resistance to chemotherapy and radiotherapy [5–7]. Similar to other cancer cells, HNSCC CSCs express surface markers such as CD44 and CD133, which are used for identification [5, 8].

Epithelial to mesenchymal transition (EMT) is important for the acquisition of CSC properties [9]. Several transcription factors, including SNAIL, TWIST, and ZEB1, have been identified as master regulators of EMT [10, 11] because they play an important role in acquisition of CSC-like properties.

MicroRNAs are small non-coding RNA molecules that regulate various aspects of cancer biology, including tumor initiation, drug resistance, and metastasis [12–14]. The miR-200 family (miR-200a, miR-200b, miR-429 miR-200c, and miR-141) suppresses the EMT phenotype by directly targeting the ZEB1 gene [11]. Since ZEB1 also negatively regulates the miR-200 family at the transcriptional level [15], the reciprocal repression between ZEB1 and miR-200 family is thought to be an important factor responsible for maintenance of CSC properties.

While a number of studies report the role of EMT in the generation and maintenance of CSCs, the mechanisms underlying acquisition of the EMT phenotype are unclear. Here, we found that CDDP induced the conversion of CD44v to CD44s in oral cancer cells, resulting in ZEB1-mediated EMT induction. Further analysis revealed that CD44s induced EMT by repressing miR-200c. Therefore, we describe a key molecular mechanism by which resistance to chemotherapy promotes acquisition of CSC properties by HNSCC cells. These findings will increase our understanding of the biological link between platinum resistance and CD44, which leads to the generation of CSCs in populations of oral cancer cells.

## RESULTS

### CDDP induces EMT in oral cancer cell lines

We first examined drug-resistant cell populations using four oral cancer cell lines: HSC-3, HSC-4, SCC-25, and SAS. Because CSCs show tumor seeding ability and resistance to conventional therapies [16], we examined expression of established CSC markers such as CD44 and aldehyde dehydrogenase (ALDH) activity [17, 18] after CDDP treatment. Because the IC_50_ values for SAS cells in response to CDDP were about 3.2 ± 0.8 µM (Supplementary Figure 1A), cells were treated with a lower concentration of CDDP (1.7 µM CDDP) for 72 h. After CDDP treatment, we performed flow cytometry analysis and found an increase in the CD44^low^ population that showed lower ALDH activity than the non-treated cells (Supplementary Figure 1B). This was also true for the other cell lines tested (HSC-3, HSC-4, and SCC-25). Therefore, contrary to our hypothesis, CDDP increased the CD44^low^ population in all four oral cancer cell lines. To understand the role of the CD44 ^low^ cell population in oral cancer biology, we next analyzed the properties of these cells. Since SAS cells contained the largest CD44^low^ population of the four cell lines after treatment with 1.7 µM CDDP, we used these cells in future experiments.

**Figure 1:**
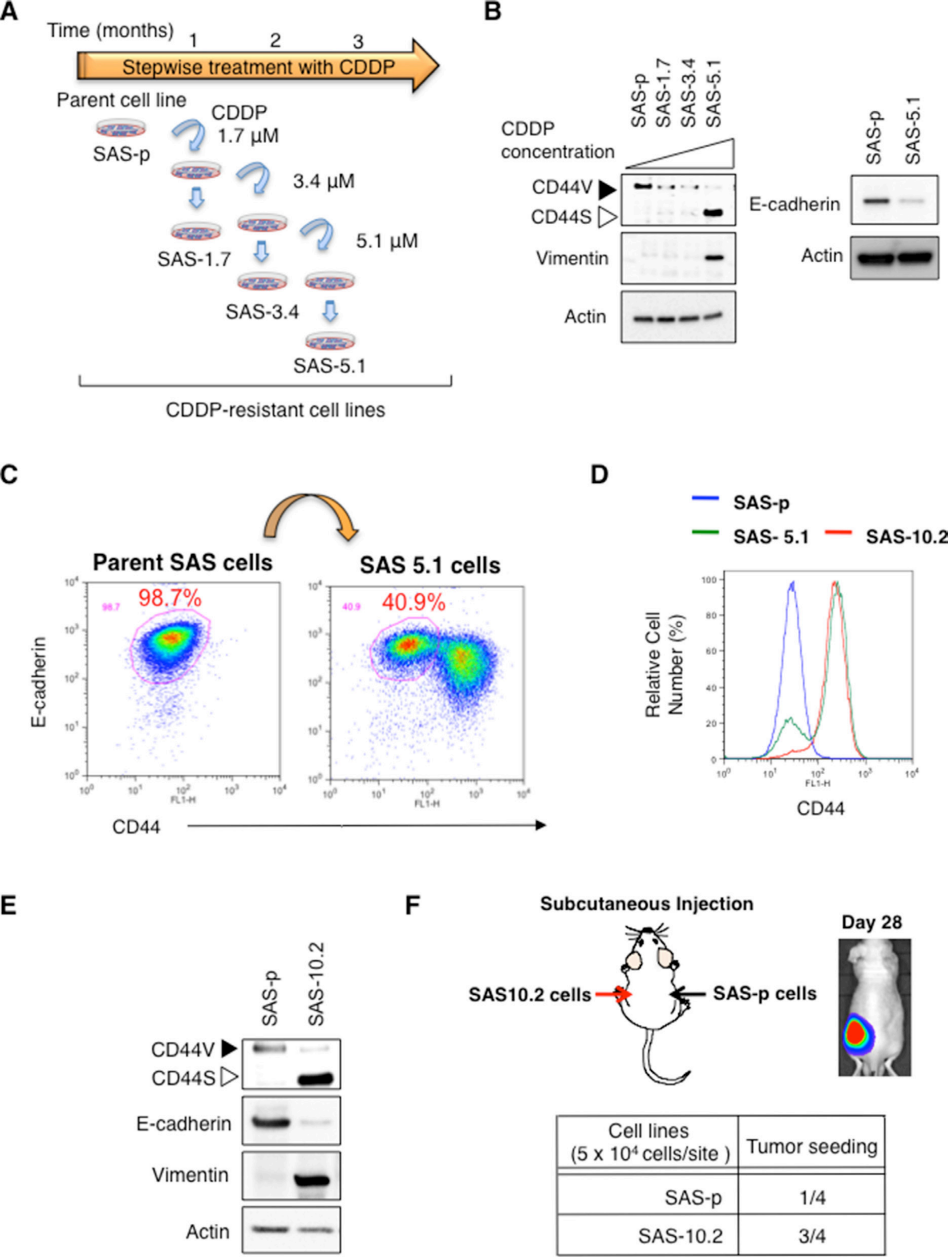
Preparation of CDDP-resistant oral cancer cell lines (**A**) Overview of the method used to prepare CDDP-resistant SAS cell derivatives. CDDP-resistant SAS derivatives were obtained by increasing the CDDP concentration from 1.7 µM to 5.1 µM each one month. (**B**) Immunoblot analysis of CD44, vimentin (vim), and E-cad expression by SAS cell derivatives. (**C** and **D**). Flow cytometry analysis of CD44 and E-cadherin expression by SAS cell derivatives. (**E**) Immunoblot analysis of CD44, vim, and E-cad expression by SAS cell derivatives. (**F**) Tumor seeding ability of CDDP resistant oral cancer cells. The number of animals with detectable tumors in groups injected with the SAS cell derivatives (Left: SAS-10.2 cells, Right: SAS-p cells).

To examine expression of CD44 in SAS cells after exposure to much higher concentrations of CDDP, we prepared CDDP-resistant SAS derivatives by increasing the CDDP concentration from 1.7 µM to 5.1 µM in a stepwise manner (Figure 1A). After preparing CDDP-resistant cell lines, we examined expression of CD44 by immunoblot analysis. CDDP treatment from 1.7 µM to 3.4 µM reduced CD44v expression by SAS cells (Figure 1B). More importantly, a high concentration of CDDP (5.1 µM) induced CD44s expression (Figure 1B). Since CD44 has multiple isoforms, which are generated via alternative splicing [19], we also investigated expression of CD44 variant forms by PCR. Consistent with the results of immunoblot analysis, PCR analysis also revealed that CDDP induced a switch in the CD44 isoform in SAS cells from CD44v to CD44s (Supplementary Figure 2A).

To investigate whether the switch from CD44v to CD44s is associated with acquisition of CSC properties, we examined induction of EMT in CDDP-resistant SAS derivatives by immunoblot analysis. Compared with SAS-p cells, SAS-5.1 cells showed down-regulated expression of E-cadherin (E-cad), an epithelial marker, and up-regulated expression of vimentin (vim), a mesenchymal marker (Figure 1B and 1C). Since the EMT phenotype promotes acquisition of CSC properties (such as drug resistance and high tumor seeding ability) in cancer cells [20], these results indicate that in the CD44 ^low^ cell population, drug-resistant CD44s-positive cells are generated from CD44v-positive cells that underwent EMT, resulting in acquisition of CSC properties.

Because SAS-10.2 cells established from SAS-5.1 cells by exposure to 10.2 µM CDDP for 1 month showed increased expression of CD44s and comprised mainly E-cad (-) cells (Figure 1D and 1E), we next examined the tumor seeding ability of SAS-10.2 cells via *in vivo* imaging. While SAS-p cells showed low tumor seeding ability in 7-week-old nude mice (BALB/cAJcl-nu/nu), the tumor seeding ability of SAS-10.2 cells was high (Figure 1F). These results again suggest that CDDP induces a switch from CD44v to CD44s in SAS-p cells, and that such cells acquire CSC properties.

### CD44s induces EMT only in CDDP-resistant SAS cells

**Figure 2:**
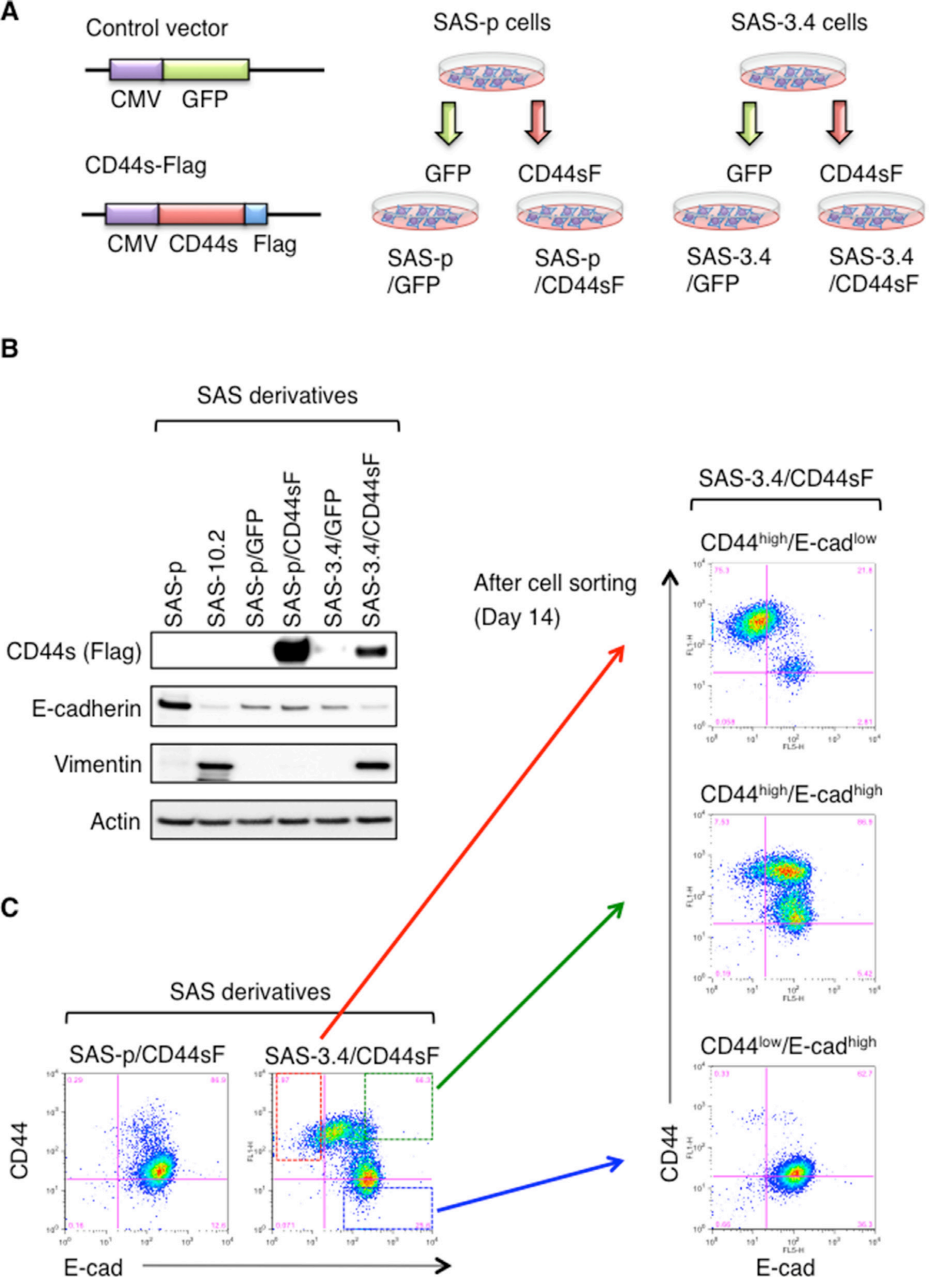
CD44s promotes EMT only in CDDP-resistant oral cancer cells (**A**) Overview of the method used to establish CD44s- or GFP-expressing SAS derivatives. The C-terminal Flag-tagged CD44 expression vector was used to prepare CD44s-expressing SAS-p and SAS-3.4 cells. The GFP-expressing construct was used as a negative control. (**B**) Immunoblot analysis of CD44s-Flag, vim, and E-cad expression by SAS-p, SAS-10.2, SAS-p/GFP, SAS-p/CD44sF, SAS-3.4/GFP, and SAS-3.4/CD44sF cells. (**C**) Flow cytometry analysis of CD44s-expressing SAS derivatives. After cell sorting of SAS-3.4/CD44sF, each fraction was cultured for 14 days and re-analyzed.

Since EMT induction and expression of CD44s were observed concomitantly in the CDDP-resistant SAS cell line SAS-5.1 (Figure 1A and 1B), we next examined whether EMT was induced by expression of CD44s in SAS cells. For this purpose, we prepared the SAS-p cells and SAS-3.4 cells stably expressing C-terminal Flag-tagged CD44s (CD44sF) (Figure 2A). After preparing the SAS derivatives (SAS-p/CD44sF and SAS-3.4/CD44sF cells), we examined expression of E-cad and vim by immunoblotting. SAS-3.4/CD44sF showed lower expression of E-cad and higher expression of vim than SAS-p/CD44sF cells (Figure 2B). Consistent with these results, flow cytometry revealed that CD44sF induced the generation of CD44^high^/E-cad^low^ cell cells (Figure 2C). While both SAS-p/CD44sF cells and SAS-3.4/CD44sF cells expressed CD44sF (Figure 2B), EMT induction by CD44s was observed only in SAS-3.4 cells. More importantly, SAS-3.4/CD44sF cells contained three cell populations: CD44^high^/E-cad^low^, CD44^high^/E-cad^high^, and CD44^low^/E-cad^high^ (Figure 2C). At Day 14 after cell sorting, CD44^high^/E-cad^low^ and CD44^high^/E-cad^high^ cells reconstituted the other cell population (CD44^low^/E-cad^high^ cells), whereas CD44^low^/E-cad^high^ cells did not (Figure 2C). This suggests that expression of CD44s is essential for induction of the EMT phenotype in CDDP-resistant oral cancer cells, resulting acquisition of the ability to undergo asymmetric cell division. Since CDDP treatment (3.4 µM for 1 month) promoted generation of the CD44^high^/E-cad^low^ cell fraction even in the SAS-p/CD44sF cell population (Supplementary Figure 3), these results also indicate that CD44s can promote the EMT phenotype only in a CDDP resistant cell population of oral cancer cells.

### The C-terminal intracellular domain (ICD) of CD44 is important for EMT induction

**Figure 3:**
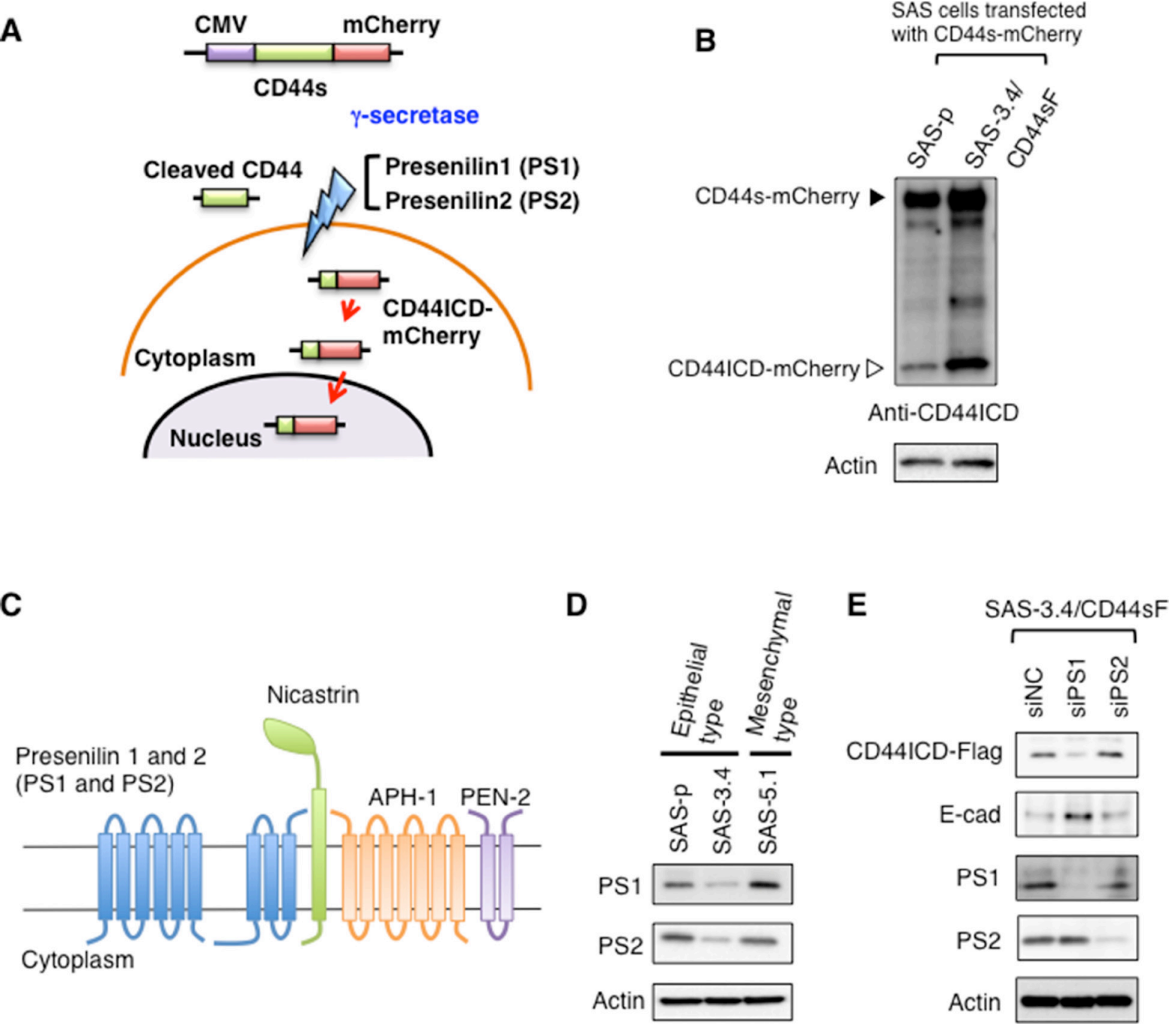
CD44ICD plays an important role in EMT induction (**A**) Schematic illustration showing C-terminal mCherry-tagged CD44s (CD44s- mCherry). CD44ICD of CD44s-mCherry was generated by γ-secretase-mediated cleavage of CD44, after which it was localized to the nucleus. (**B**) Immunoblot analysis of CD44ICD in SAS derivatives after expression of CD44-mCherry. (**C**) γ-secretase mainly comprises PS1, PS2, nicastrin, APH-1, and PEN-2. (**D**) Immunoblot analysis of PS1 and PS2 expression by SAS-p, SAS-3.4, and SAS-5.1 cells. (**E**) Knockdown of PS1 and PS2 in CD44sF-expressing SAS-3.4 cells. Immunoblot analysis of PS1, PS2, CD44ICD, E-cad, and vim expression by SAS-3.4/CD44sF cells.

Since CD44s induced acquisition of the EMT phenotype only in CDDP-resistant oral cancer cells (Figure 2), we next investigated the mechanism underlying CD44s-meditated EMT induction. The CD44ICD acts as an intracellular signaling molecule [21, 22]; therefore, we hypothesized that the amount of CD44ICD would be specifically increased in CDDP-resistant SAS cells, resulting in EMT (Figure 2). Several studies report that after cleavage of CD44 into extracellular and ectodomains by membrane-associated matrix metalloproteinases such as MT1-MMP and ADAM10, CD44ICD is generated from via subsequent cleavage of the CD44 ectodomain by presenilin (PS)-dependent γ-secretase [23] (Figure 3A). After the second cleavage of CD44 ectodomain, cytoplasmic CD44ICD is transported to the nucleus where it transcriptionally activates various genes, including *CD44* [23] (Figure 3A). To measure the amount of CD44ICD in SAS-3.4/CD44sF cells, we prepared C-terminal mCherry-tagged CD44s (CD44s-mCherry) and transiently expressed it in SAS-p and SAS-3.4/CD44sF cells (Figure 3B). Immunoblot analysis revealed that SAS-3.4/CD44sF cells expressed higher levels of CD44ICD than SAS-p cells (Figure 3B).

γ-secretase mainly comprises five proteins: PS (including PS1 and PS2), nicastrin, anterior pharynx defective 1 (APH-1), and presenilin enhancer 2 (PEN2); PS1 and PS2 are the catalytic subunits responsible for the aspartic protease activity of the γ-secretase complex [24] (Figure 3C). Therefore, we next examined expression of PS1 and PS2 in SAS-p, SAS-3.4 and SAS-5.1 cells by immunoblot analysis and observed higher expression of PS1 in SAS-5.1 cells than in SAS-p and SAS-3.4 cells (Figure 3D). To examine the roles of PS1 in CD44s-mediated EMT induction, we knocked down PS1 and PS2 in SAS-3.4/CD44sF cells. Immunoblot analysis revealed that only knockdown of PS1 reduced the amount of CD44ICD and suppressed the EMT phenotype (Figure 3E). Therefore, these results suggest that PS-dependent γ-secretase-mediated cleavage of CD44s is essential for induction of EMT in CDDP-resistant oral cancer cells.

### CD44s induces the EMT phenotype in CDDP-resistant oral cancer cells by regulating the feedback loop between miR-200c and ZEB1 expression

**Figure 4:**
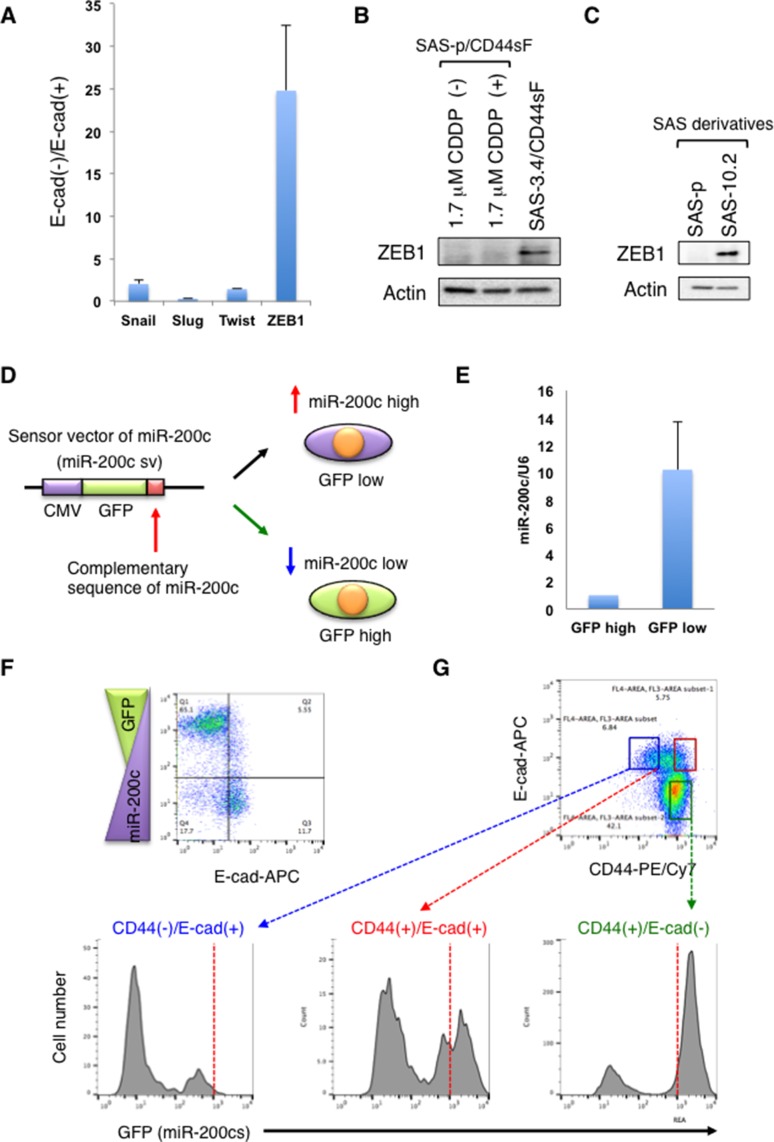
CD44s induces ZEB1 expression by suppressing miR-200c expression (**A**) Expression of EMT regulators by the E-cad (+) and E-cad (−) fractions within the SAS-3.4/CD44sF cell population. (**B** and **C)**. Immunoblot analysis of ZEB1 protein expression by SAS derivatives. (**D**) Schematic illustration of the sensor vector harboring miR-200c. (**E**) Expression of miR-200c in miR-200c sv-expressing SAS-3.4/CD44sF cells. After cell sorting, the expression levels of miR-200c in GFP(−) and GFP(+) fractions were examined. (**F**) Relationship between GEP and E-cad expression in miR-200c sv-expressing SAS-3.4/CD44sF cells. (**G**) Flow cytometry analysis of miR-200c sv-expressing SAS-3.4/CD44sF cells. The expression level of miR-200c in each fraction was evaluated using the miR-200c sensor vector.

To further investigate the mechanism by which CD44s induces the EMT phenotype in SAS-3.4 cells, we examined expression of EMT regulators (*SNAIL, SLUG, TWIST, ZEB1*) by quantitative RT-PCR (qRT-PCR) after sorting E-cad (+) and E-cad (-) cells from the SAS3.4/CD44sF cell population. QRT-PCR analysis revealed that ZEB1 expression in E-cad (-) cells was 25 times higher than that in E-cad (+) cells (Figure 4A). Consistent with these results, we observed higher expression of ZEB1 protein in SAS3.4/CD44sF cells than in parent cells expressing CD44s (Figure 4B). Moreover, SAS-10.2 cells showed higher expression of ZEB1 than SAS-p cells (Figure 4C). These results indicate that CD44sF induces the EMT phenotype in CDDP-resistant oral cancer cells by up-regulating ZEB1 expression.

Next, we investigated expression of miR-200c in SAS-3.4/CD44sF cells. Since reciprocal repression of miR-200c and ZEB1 promotes EMT in several types of cancer cell [15], we hypothesized that CD44s first suppresses expression of miR-200c in SAS-3.4 cells, thereby up-regulating ZEB1. To evaluate expression of miR-200c in CD44^high^/E-cad^high^, CD44^low^/E-cad^high^, and CD44^high^/E-cad^low^ cell fractions derived from SAS-3.4/CD44sF cells, we prepared a sensor vector of miR-200c corresponding to a GFP-expressing construct containing the complementary sequence of miR-200c in its 3’UTR (miR-200c sv; Figure 4D). As expected, qRT-PCR combined with cell sorting revealed that expression of miR-200c in GFP-negative cells was about 10 times higher than that in GFP-positive cells (Figure 4E). In addition, we confirmed the inverse correlation between GFP and E-cad expression in SAS-3.4/CD44sF expressing miR-200c sv (Figure 4F). Using this construct, we then examined the relationship between miR-200c, CD44s, and E-cad in SAS-3.4/CD44sF cells. Flow cytometry analysis showed that CD44^high^/E-cad^high^ and CD44^high^/E-cad^low^ fractions expressed higher levels of GFP than the CD44^low^/E-cad^high^ fraction (Figure 4G). As CD44s induces GFP expression even in E-cad (+) cells, these results suggest that CD44s up-regulates ZEB1 in CDDP-resistant oral cancer cells by suppressing miR-200c.

### Expression of CD44s and EMT markers by established oral cancer cell lines

**Figure 5:**
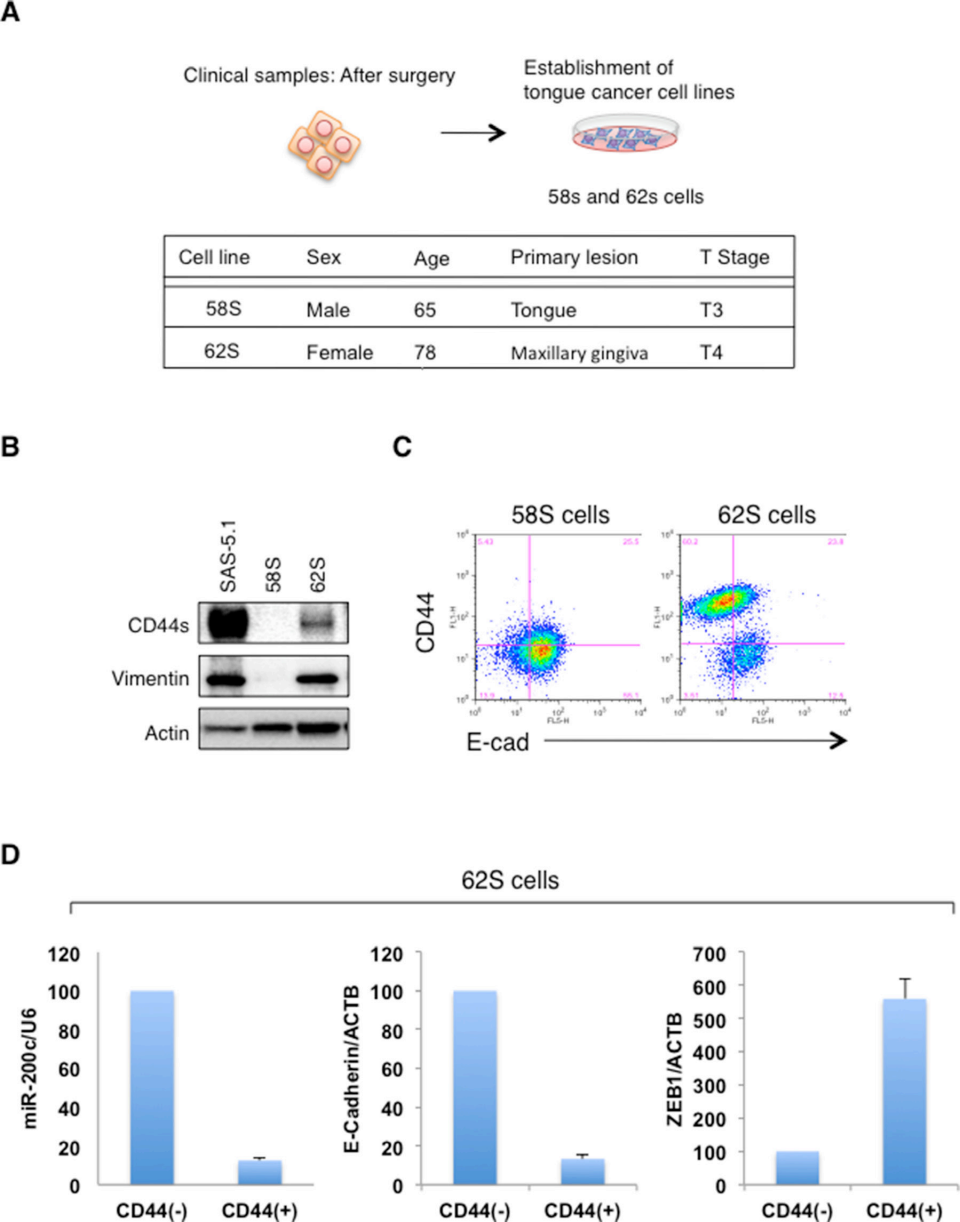
Evaluation of CD44s and EMT markers in the established oral cancer cells (**A**) Overview of the method used to establish oral cancer cells (58S and 62 cells) from oral cancer patients. (**B**) Immunoblot analysis of CD44s, vim, and E-cad expression in 52S, 68S, and SAS-5.1 cells. (**C**) Flow cytometry analysis of CD44 and E-cad expression in 52S and 68S cells. (**D**) Expression of miR-200c and ZEB1 in 62S cells. After cell sorting, the expression levels of miR-200c and ZEB1 in CD44(+) and CD44(-) fractions were examined.

To confirm the relationship between CD44s and EMT markers, we examined the expression of CD44s, vim, and E-cad in two oral cancer cell lines (58S and 62S) established from oral cancer specimens obtained from patients at our hospital (Figure 5A). Immunoblot analysis revealed that 62S cells showed higher expression of CD44s and vim than 58S cells (Figure 5B). Flow cytometry analysis also revealed that the CD44s-positive cell population contained E-cad-negative cells (Figure 5C). More importantly, qRT-PCR analysis coupled with cell sorting revealed that the expression levels of miR-200c and E-cadherin were significantly lower in the CD44s positive fraction than in the CD44s negative fraction in 62S cells (Figure 5D). We also observed elevated expression of ZEB1 in the CD44s positive fraction. Therefore, consistent with the results shown in Figure 1, these results suggest that EMT mainly occurs in CD44s-expressing oral cancer cells.

### CD44s exerts a functional role in TGF-β1-mediated EMT induction

**Figure 6:**
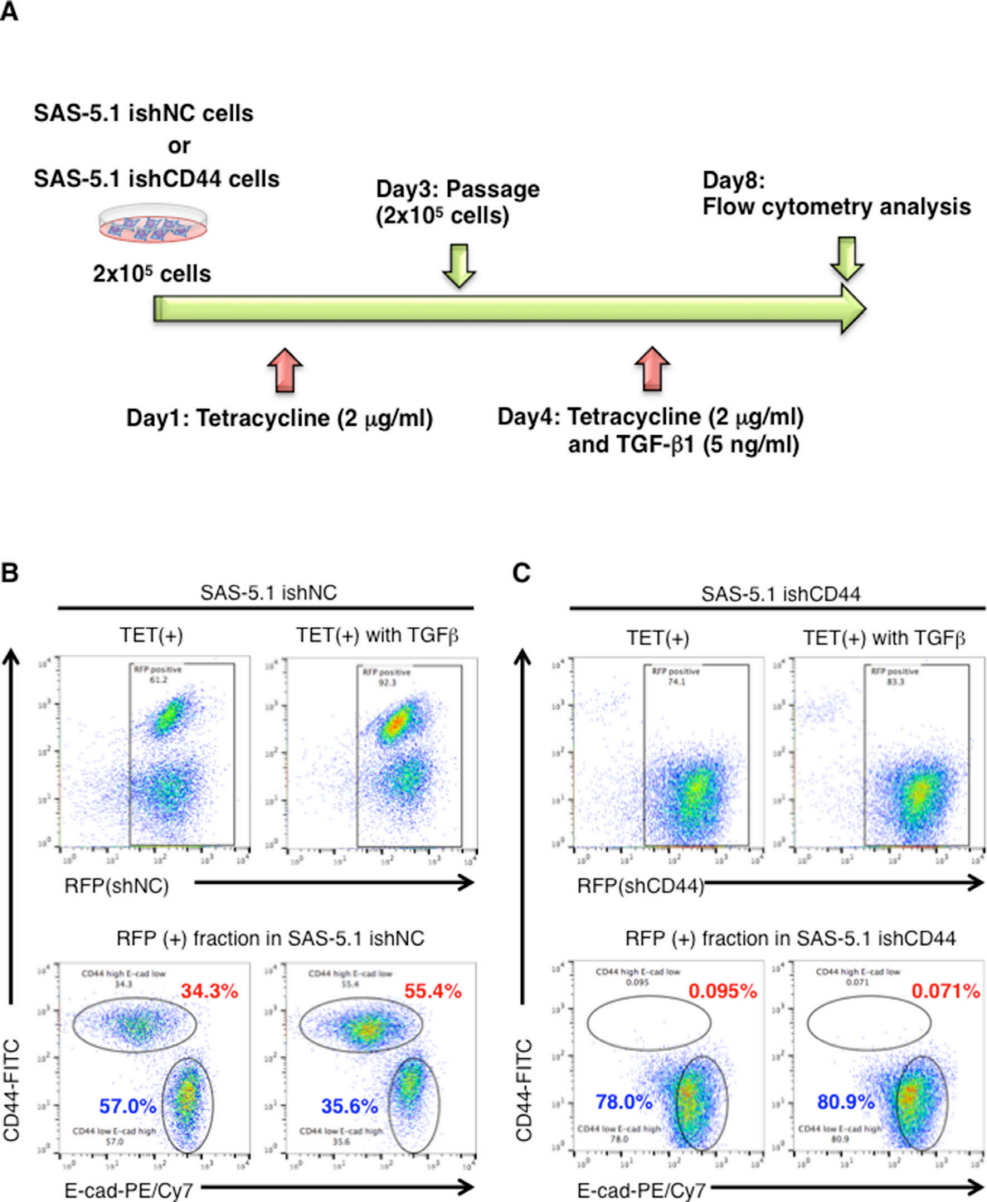
CD44s plays an important role in TGB-β1-mediated EMT in CDDP resistant oral cancer cells (**A**) Schematic representation of tetracycline inducible CD44s knockdown in SAS-5.1 cells. CD44s was first knock-downed by tetracycline (TET) treatment (2 μg/ml) for 96 h in SAS-5.1 ishCD44 cells, and then EMT was induced by TGF-β1 treatment for 96 h under CD44s knockdown conditions. (**B**) The expression of RFP and non-targeting shRNA or shRNA against CD44s was induced by TET treatment (2 μg/ml) in SAS-5.1 ishNC cells (Left panel). (**C**) The expression levels of CD44s and E-cad were evaluated in SAS-5.1 ishNC cells after TET (2 μg/ml) and TGF-β1 treatment (5 ng/ml) (Right panel). The expression of RFP and non-targeting shRNA or shRNA against CD44s was induced by TET treatment (2 μg/ml) in SAS-5.1 ishCD44s cells (Left panel). The expression levels of CD44s and E-cad were evaluated in SAS-5.1 ishCD44s cells after TET (2 μg/ml) and TGF-β1 treatment (5 ng/ml) (Right panel).

**Figure 7:**
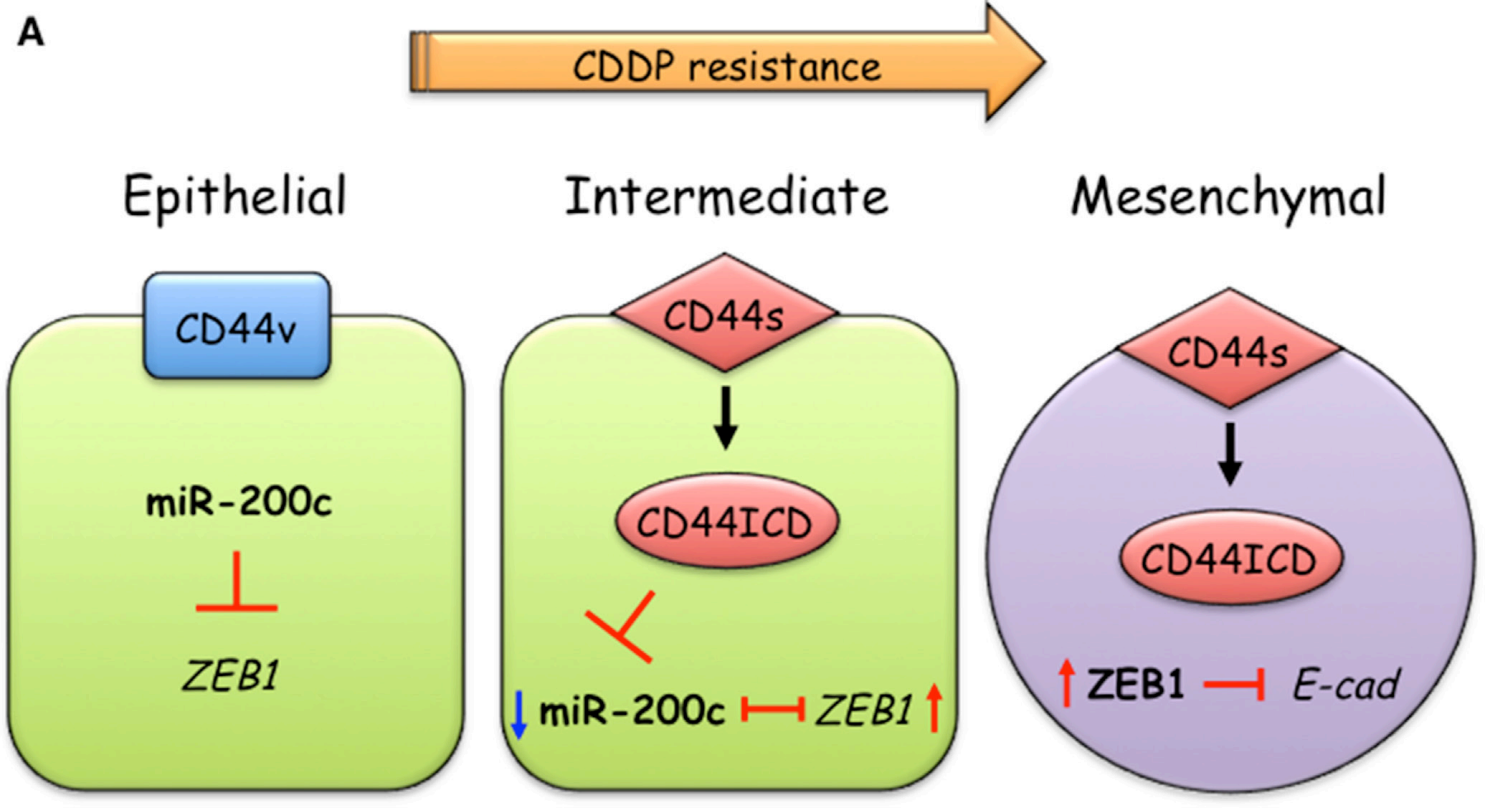
A model of CD44s-mediated EMT induction in CDDP-resistant oral cancer cells CDDP-sensitive cells mainly express CD44 variant forms, thereby maintaining epithelial status. During acquisition of CDDP resistance, switching from CD44v to CD44s and elevated expression of PS1 induces the generation of the CD44 intracellular domain (CD44ICD). CD44ICD then promotes ZEB1-mediated EMT by suppressing miR-200c.

Finally, we examined whether TGF-β1 treatment induces the EMT phenotype via the CD44s-mediated pathway. For this analysis, we established CD44 knockdown SAS-5.1 cells using the tetracycline inducible knockdown system in which RFP and shRNA against CD44 are expressed in the presence of tetracycline (Figure 6A). After tetracycline treatment, CD44s expression was markedly lower in the RFP positive fraction of SAS-5.1 ishCD44 cells than in the RFP positive fraction of control cells (SAS-5.1 ishNC cells) (Figure 6B and 6C, upper left panels). More importantly, we observed that CD44 knockdown efficiently inhibited TGF-β1-mediated EMT. While TGF-β1 treatment induced about a 2-fold increase in E-cadhein(−)/CD44(+) cells in the RFP positive fraction of SAS-5.1 ishNC cells, TGF-β1 treatment did not induce E-cadhein(−)/CD44(+) cells in the RFP positive fraction of SAS-5.1 ishCD44 cells (Figure 6B and 6C, lower panels). Therefore, these results suggest that the CD44s-ZEB1 pathway plays an important role in TGF-β1-mediated EMT induction (Figure 7A).

## DISCUSSION

While CSCs and their specific surface markers have been identified in many types of cancer, the roles of CSC markers in acquisition of CSC-like properties such as chemo-resistance and EMT induction are unclear. Here, we found that HNSCCs acquired resistance to CDDP via conversion of CD44v to CD44s. More importantly, we demonstrated that CD44s induced ZEB1-mediated EMT induction in CDDP-resistant HNSCCs by suppressing miR-200c expression. Therefore, our findings reveal a key role for CD44 during acquisition of CSC-like properties by HNSCC.

A recent study reports that switching from CD44v to CD44s is important for acquisition of the EMT phenotype in breast cancer cells, leading to cancer progression [25]. In breast cancer, CD44s supports cell survival after TGF-β or Twist-mediated induction of EMT by enhancing Akt activation [25–27]. Here, we identified another important pathway of EMT induction that is mainly initiated via the CD44s-miR-200c-ZEB1 cascade. Since CD44s-mediated induction of EMT occurred only in CDDP-resistant HNSCCs and CD44s was not localized to the cell surface in CDDP-sensitive HNSCCs, our findings suggest that CD44s-mediated induction of EMT mainly occurs during conventional chemotherapy. These results also prompted us to consider the possibility that some molecules associated with CDDP resistance support CD44s function, and that CD44s is involved in regulating miRNA biogenesis. Therefore, future studies should identify putative co-factors and elucidate the mechanisms by which CD44s regulates miRNA expression in CDDP-resistant HNSCCs. Our study also revealed that PS1 plays an important role in CD44s-mediated EMT induction. Therefore, in addition to confirming previous studies showing that gamma-secretase inhibitors are effective in inhibiting CSC properties via suppression of Notch activity [28, 29], the present results point to the existence of another mechanism mediating the suppressive effects of gamma-secretase on CSCs. Combination treatment using CDDP and gamma-secretase inhibitors might be a useful approach in overcoming the resistance of several types of cancers to chemotherapy.

In conclusion, the findings presented herein will increase our understanding of the biological functions of CSC markers during acquisition of CSC-like properties. Since CD44s supports the induction of EMT and acquisition of CDDP resistance, our findings suggest that suppressing of CSC markers may inhibit the development of malignant tumors and improve therapeutic outcomes for HNSCC patients.

## MATERIALS AND METHODS

### Antibodies

The following primary antibodies (and dilution factors) were used: anti-CD44 (1:1000; 3570, CST), anti-CD44-ICD (1:1000; KO601, TransGenetic), anti-Vimentin (1:2000; 550513, BD Biosciences), anti-E-cadherin (1:2000; 3195, CST), anti-ZEB1 (1:1000, 3396, CST), anti-Presenilin-1 (1:1000, 5643, CST), anti-Presenilin-2 (1:1000, CST, 9979), anti-Flag (1:2000; PM053-7, MBL), and anti-actin (1:5000; PM053-7, MBL).

### Plasmids

To generate vectors for expression of CD44s, *CD44s* cDNA was cloned into the multi-cloning sites of the p3XFLAG-CMV-14 expression vector (Sigma) driven by the CMV promoter. To prepare the expression vector harboring C-terminal mCherry-tagged CD44s, *CD44s* cDNA was inserted into pmCherry-N1 (Clontech). To prepare the lentiviral vector expressing firefly luciferase, an amplified PCR fragment of firefly luciferase was inserted into pCDH-CMV-MCS-EF1-Greenpuro (System Biosciences). To prepare miR-200c sv, a single complementary sequence of miR-200c was inserted into the 3’UTR of pEGFP-N1 (Clontech).

### Cell culture

HSC-3, HSC-4, and SAS cells were obtained from the Japanese Collection of Research Bioresources Cell Bank, and SCC-25 cells were obtained from ATCC. HSC-3 and SCC-25 cells are human oral squamous carcinoma cell lines with high metastatic potential [30, 31]. All four cell lines are human oral squamous carcinoma cell lines derived from tongue tissues and show an epithelial-like morphology. Each cell line was grown under the culture conditions recommended by the depositors. To prepare CDDP-resistant SAS derivatives, cells were cultured in the presence of increasing concentrations of CDDP from 1.7 to 10.2 µM each month (Maruko).

### Real-time RT-PCR

Total RNA and miRNA were isolated from cells using the RNeasy Mini Kit (QIAGEN), and cDNA produced using the ExScript RT reagent Kit (Takara) or the TaqMan MicroRNA Reverse Transcript Kit (Applied Biosystems) according to the manufacturer’s instructions. TaqMan probes were obtained from Applied Biosystems. The cDNA samples were subjected to real-time PCR using SYBR Premix Ex Taq (Takara). The specific primers are listed in Supplementary Table 1. TaqMan MicroRNA Assays were used for qRT-PCR analyses of miRNAs (Applied Biosystems). Reactions were performed on the StepOnePlus Real-Time PCR System (Applied Biosystems). Expression levels were normalized to those of *GAPDH* or *RNU6B*, and relative expression was calculated using the 2^ΔΔCt^ method.

### Fluorescence-activated cell sorting

An APC-, FITC-, or PE/Cy7-conjugated anti-CD44 antibody (BD Biosciences, clone G44-26), an APC-conjugated anti-CD4 antibody (BD Biosciences, clone SK3), an APC- or PE-Cy7-conjugated anti-E-cadherin antibody (BD Biosciences, clone 67A4) and propidium iodide (5 μg/ml; BD Biosciences) were used for fluorescence-activated cell sorting analyses. Flow cytometric analysis and cell sorting were performed using a JSAN cell sorter (Bay bioscience) and an S3 cell sorter (Bio-Rad) and data analyzed with FlowJo software.

### Bioluminescence imaging

All animal experiments were performed in accordance with the guidelines of the Institute for Laboratory Animal Research, National Cancer Center Research Institute. Male nude mice (BALB/cAJcl-nu/nu; CLEA Japan) aged 4–7 weeks were used for the xenograft model. Images were analyzed with Living Image software (Caliper Life Sciences).

### Patients samples

The study was approved by the Institutional Review Board of Showa University (No. 8) and informed consent was obtained from all patients for the establishment of oral cancer cell lines from patient tissues.

### Statistical analysis

Data are expressed as the mean ± SD. Unless stated otherwise, statistical significance was determined using Student’s two-tailed *t-*test. *P* < 0.05 was considered statistically significant.

## SUPPLEMENTARY MATERIALS FIGURES AND TABLE



## Abbreviations

HNSCC: head and neck squamous cell carcinoma
CSC: Cancer stem cell
miRNA: microRNA
EMT: epithelial to mesenchymal transition
E-cad: E-cadherin
vim: vimentin
CD44s: CD44 standard form
CD44v: CD44 variant form
PS: presenilin
