# Supplementary material for: CD44 exerts a functional role during EMT induction in cisplatin-resistant head and neck cancer cells
Source: Oncotarget. 2018 Jan 13;9(11):10029–41. doi: 10.18632/oncotarget.24252 (PMC5839369; doi:10.18632/oncotarget.24252)
Supplement: Supplementary file 1 [file oncotarget-09-10029-s001.pdf]

CD44 exerts a functional role during EMT induction in cisplatin-resistant head and neck cancer cells

SUPPLEMENTARY MATERIALS

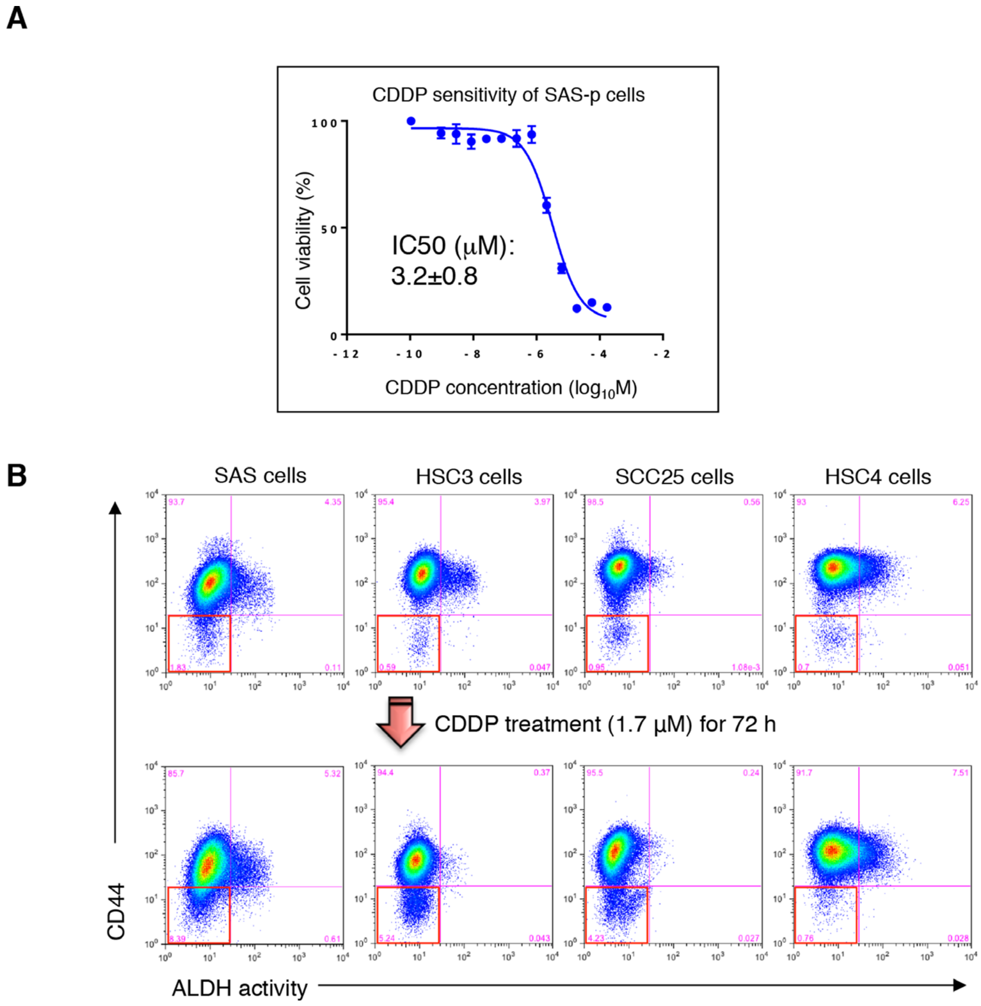

Supplementary Figure 1: Flow cytometry analysis of CDDP-treated oral cancer cells. (**A**) The IC50 values for CDDP when used to treat SAS cells. (**B**) Expression of CD44 and ALDH activity by CDDP-treated cells.

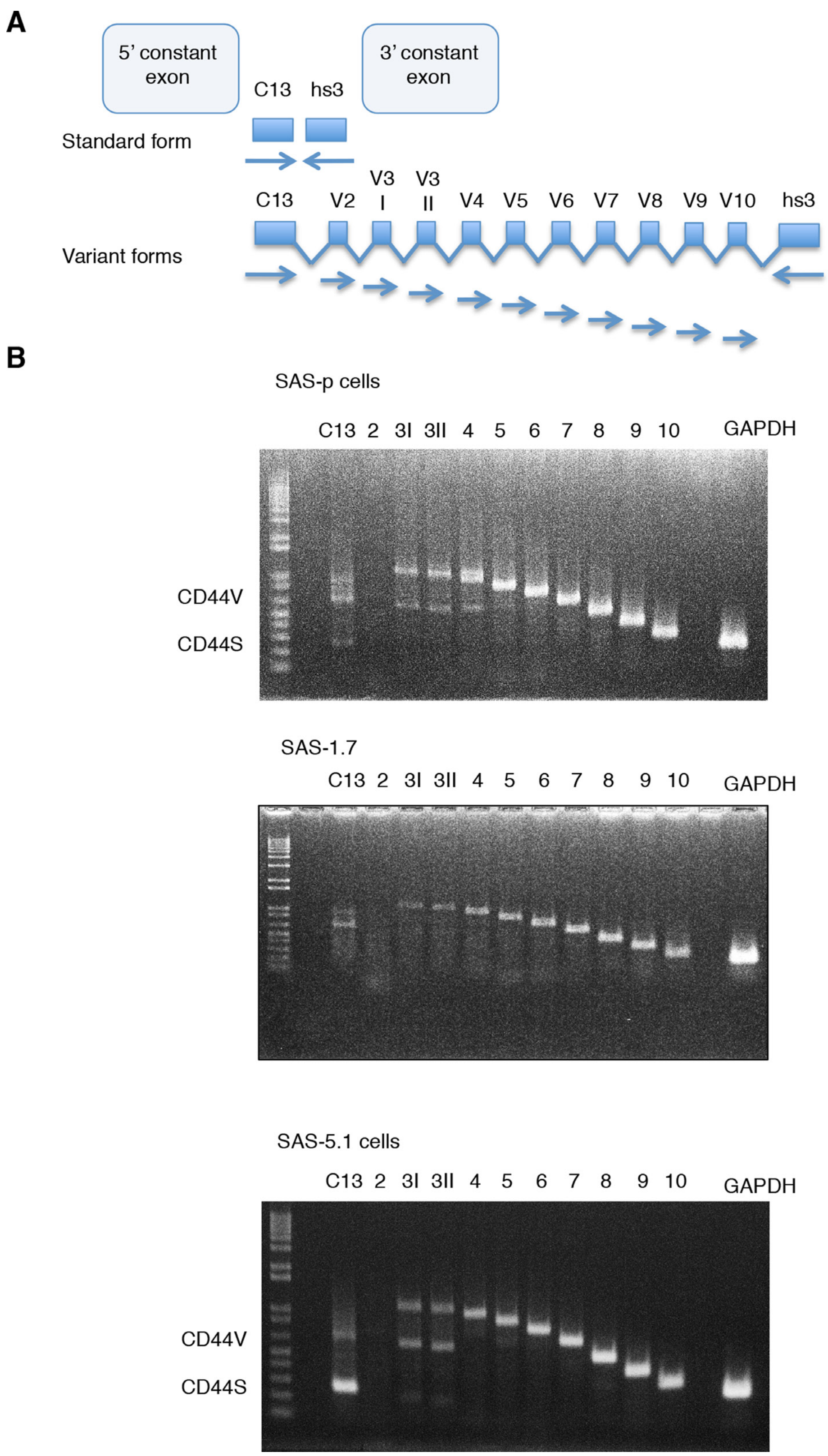

Supplementary Figure 2: PCR analysis of CD44 isoforms expressed by SAS derivatives. (**A**) A schematic illustration of the specific primers used to amplify CD44 isoforms. (**B**) Expression of CD44 isoforms by SAS derivatives.

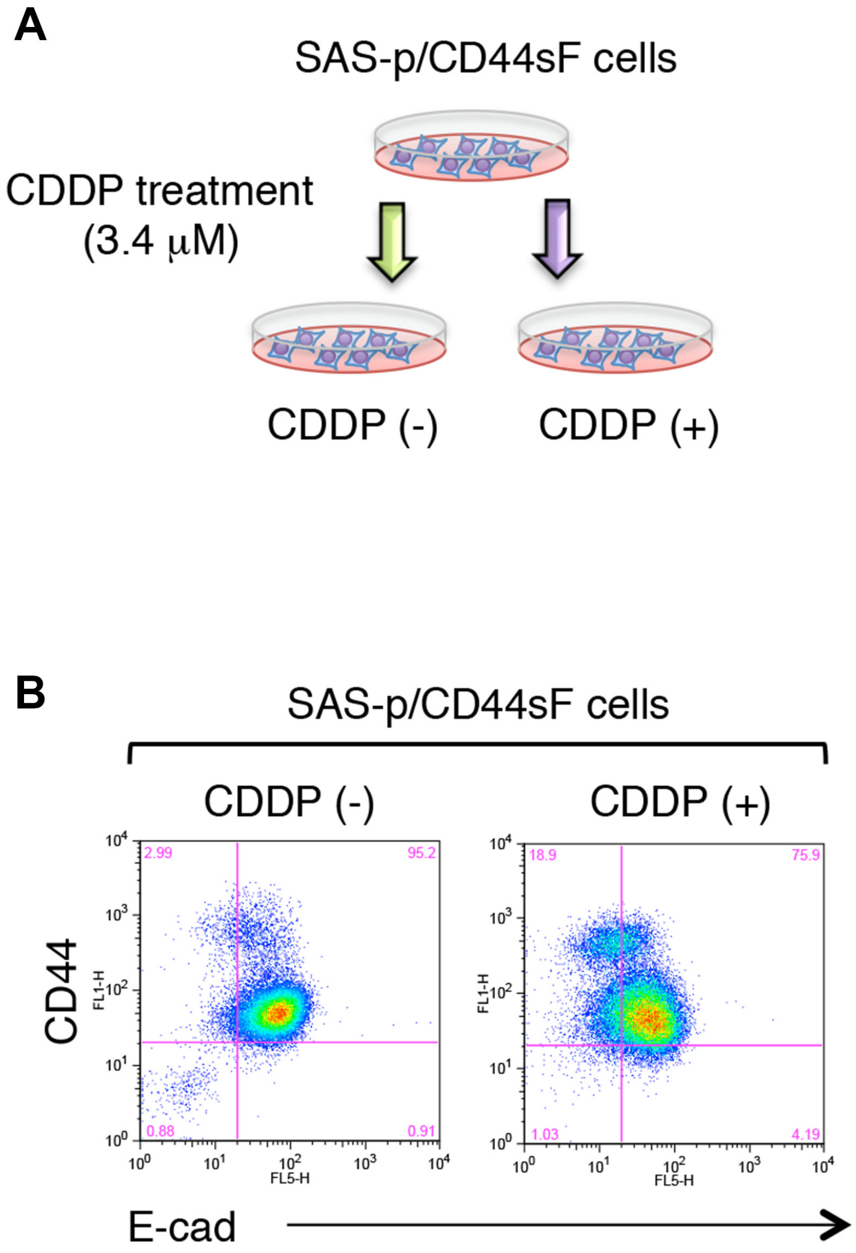

Supplementary Figure 3: CDDP treatment induces cell surface localization of CD44s in SAS cells. (**A**) Overview of the method used to establish CD44s-expressing SAS-p cells. (**B**) Localization of CD44s in SAS cells after exposure to 3.4 μM CDDP.

Supplementary Table 1: Primer sequence used for this study

Primer name	Nucleotide sequence
hACTB Fw	5′-ggacttcgagcaagagatgg-3′
hACTB Rv	5′-agcactgtgttggcgtacag-3′
hZEB1 Fw	5′-accagacagtgttaccaggg-3′
hZEB1 Rv	5′-acatcctgcttcatctgcct-3′
hSnail Fw	5′-gcgagctgcaggactctaat-3′
hSnail Rv	5′-ggacagagtcccagatgagc-3′
hSlug Fw	5′-ctttttcttgccctcactgc-3′
hSlug Rv	5′-agcagccagattcctcatgt-3′
hTwist Fw	5′-ggagtccgcagtcttacgag-3′
hTwist Rv	5′-ccagcttgagggtctgaatc-3′
